# Risque résiduel de la transmission par transfusion de l´infection du virus de l´hépatite B dû aux donneurs porteurs de l´infection du virus de l´hépatite B occulte à Yaoundé, Cameroun

**DOI:** 10.11604/pamj.2021.39.175.22365

**Published:** 2021-07-06

**Authors:** Michel Kengne, Yolande Francine Onomo Medja, Tedom, Julius Mbekem Nwobegahay

**Affiliations:** 1 Département de Microbiologie et Immunologie Médicale, Ecole des Sciences de la Santé, Université Catholique d´Afrique Centrale, Yaoundé, Cameroun,; 2 Centre de Biotechnologie, Yaoundé, Cameroun,; 3 Centre de Recherche pour la Santé des Armées (CRESAR), Yaoundé, Cameroun

**Keywords:** Risque résiduel, VHB, infection, transfusion, transmission, Residual risk, HBV, infection, transfusion, transmission

## Abstract

**Introduction:**

l´infection virale au virus de l´hépatite B reste la plus importante transmise par transfusion sanguine. Bien que la recherche de l´antigène de surface (AgHBs) du virus de l´hépatite B (VHB) dans les banques de sang ait considérablement réduit le risque de transmission de l´infection du virus par transfusion, il subsiste un risque résiduel transfusionnel de transmission dû aux donneurs porteurs d´une hépatite B occulte. Les poches de sang contenant l´AcHBc avec ou sans l´AcHBs et l´ADN viral peuvent être infectieuses et représenter une menace pour la sécurité transfusionnelle là où l´AcHBc n´est pas dépisté. L´objectif de cette étude était de déterminer le risque résiduel de la transmission par transfusion du virus de l´hépatite B à l´hôpital central de Yaoundé (HCY) et le Centre Hospitalier Dominicain Saint Martin de Porres (CHDSMP) à Yaoundé au Cameroun.

**Méthodes:**

une étude transversale a été effectuée sur des donneurs de sang de l´Hôpital Central de Yaoundé (HCY) et le Centre Hospitalier Dominicain Saint Martin de Porres (CHDSMP) chez lesquels la recherche de l´AcHBc et/ou AcHBs était faite par immunochromatographie. La recherche de l´ADN du VHB sur les échantillons testés positifs aux AcHBc et/ou AcHBs était faite par PCR en utilisant des amorces spécifiques.

**Résultats:**

sur les 193 donneurs de sang négatifs aux marqueurs sérologiques des infections à VIH, VHB (AgHBs), VHC et tréponème retenus dans cette étude, nous avons obtenu une séroprévalence globale des AcHBc et/ou AcHBs de 9,84% (19/193). Sur les 19 donneurs potentiellement infectieux, l´ADN du virus a été détecté chez 03 individus dont 02 porteurs de l´AcHBc et 01 porteur à la fois de l´AcHBc et de l´AcHBs, soit une prévalence de l´hépatite B occulte de 15,79% (3/19) [IC95% =3,38%-39,58%] et un risque résiduel de transmission par transfusion de l´infection à VHB de 1,55% (3/193) [IC95% = 0,32%-4,48%].

**Conclusion:**

il ressort de cette étude que le risque résiduel de transmission par transfusion sanguine de l´infection du VHB est faible. Toutefois il est recommandé de rechercher les AcHBc et/ou AcHBs lors de la qualification des dons de sang.

## Abstract (en)

**Introduction:**

viral infection caused by hepatitis B virus is the most frequent transfusion-transmitted viral infection. Although the search for hepatitis B surface antigen (HBsAg) in blood banks has significantly reduced the risk for transfusion-transmitted virus infection, there is still a residual transfusion risk of transmission from donors with occult hepatitis B. Blood bags containing aHBc with or without aHBs and viral DNA can cause infections and represent a threat to transfusion safety when aHBc levels are undetectable. The purpose of this study is to determine the residual risk for transfusion-transmitted hepatitis B virus at the Central Hospital of Yaoundé (CHY) as well as at the St Martin de Porres’s Catholic Hospital (SMPCH) in Yaoundé, Cameroon.

**Methods:**

we conducted a cross-sectional study among blood donors at the Central Hospital of Yaoundé (CHY) and the St Martin de Porres’s Catholic Hospital. In these subjects the search for aHBc and/or the aHBs was conducted by immunochromatography. HBV DNA test was performed on blood samples tested positive for aHBc and/or aHBs by Polymerase Chain Reaction (PCR) technique using specific primers.

**Results:**

out of a total of 193 blood donors negative for HIV, HBV (HBsAg), HCV serological markers and treponema infections, the overall seroprevalence of aHBc and/or aHBs was 9,84% (19/193). Out of a total of 19 potentially infected donors, HBV DNA was detected in 03 individuals, including 02 aHBc carriers and 01 carrier of both aHBc and aHBs, reflecting a prevalence of occult hepatitis B of 15,79% (3/19) [IC 95% =3,38%-39,58%] and a residual risk for transfusion-transmitted hepatitis B virus of 1,55% (3/193) [IC 95% =0,32%-4,48%].

**Conclusion:**

this study shows that the residual risk for transfusion-transmitted hepatitis B virus is low. However, it is recommended to screan blood donors for aHBc and/or aHBs.

## Introduction

L´hépatite B est « une infection virale aiguë du foie qui peut devenir chronique et être à l´origine d´une cirrhose et d´un cancer primitif du foie. Elle est causée par un virus qui porte le même nom: le virus de l´hépatite B (VHB) [1]. Dans le monde on estime à environ 2 milliards les personnes ayant été en contact avec ce virus à un moment de leur vie, et 257 millions d´entre elles restent infectées de manière chronique [2]. La prévalence de l´hépatite B est plus élevée en Afrique subsaharienne et en Asie de l´Est où entre 5 et 10% de la population adulte est atteinte d´hépatite B chronique [3]. Si le risque de transmission par transfusion sanguine des virus dits majeurs (VIH, VHB, VHC) a fortement baissé dans les pays développés, il reste important dans les pays aux structures sanitaires moins développées et de forte endémicité pour ces virus [4]. L´infection virale à VHB reste la plus importante transmise par transfusion sanguine [5]. Ce risque de transmission par transfusion sanguine du VHB semble être principalement lié à des dons de sang négatifs en antigène de surface du VHB (AgHBs), mais contenant des taux extrêmement faibles d´ADN viral potentiellement infectieux. Cette transmission serait liée aux donneurs porteurs d´une hépatite B occulte (HBO) [6]. L´HBO est caractérisée par la détection des anticorps dirigés contre le noyau viral (AcHBc) dans 80% des cas [7]. La contamination du VHB par transfusion n´est plus d´actualité dans les pays développés car le dépistage de l´AgHBs et du génome viral est maintenant appliqué sur les dons de sang et d´organes [8].

Dans les zones de forte endémie telle que le Cameroun où la recherche de l´anticorps anti-Hbc n´est pas systématique chez les donneurs de sang, il subsiste donc un risque résiduel de transmission par transfusion sanguine de l´infection au VHB. Ce qui rend ainsi la transfusion sanguine dangereuse pour les receveurs de sang. Une étude menée à la banque de sang de l´Hôpital Laquintinie à Douala a révélé une séroprévalence globale de l´AcHBc de 57% chez des donneurs testés AgHBs négatifs [9]. C´est pourquoi, il est important d´estimer le risque de transfusion afin d´optimiser les stratégies de recrutement des donneurs et de minimiser la transmission de l´infection par le VHB. Cette étude a donc été menée afin de déterminer le risque résiduel de la transmission par transfusion de l´infection du virus de l´hépatite B dû aux donneurs porteurs de l´infection du virus de l´hépatite B occulte à Yaoundé au Cameroun. De cet objectif général nous avons formulé l´hypothèse de recherche suivante: quel est le risque résiduel transfusionnel de l´hépatite B sous forme occulte dans deux structures sanitaires de la ville de Yaoundé? Les objectifs spécifiques suivants ont ainsi été définis: a) déterminer la prévalence des anticorps anti-VHB (AcHBc et ou AcHBs) chez les donneurs de sang éligibles au don dans deux structures sanitaires de la ville de Yaoundé et b) identifier par PCR, l´ADN du VHB dans les échantillons des donneurs de sang AcHBc et/ou AcHBs positifs.

## Méthodes

### Type d´étude, participant et période

Nous avons réalisé une étude transversale et prospective dans les banques de sang de HCY et CHDSMP de Yaoundé qui accueillent de nombreux donneurs de sang de la cité capitale. L´enquête a duré cinq (05) mois allant d´août à décembre 2019. La population d´étude était constituée de donneurs de sang consentant et testés négatifs par le laboratoire de qualification biologique de la banque de sang aux marqueurs sérologiques AgHBs, AcVHC, AcVIH, TPHA des infections à VHB, VHC, VIH et tréponème. Les données sociodémographiques des donneurs étaient obtenues au moyen d´un questionnaire.

### Calcul de la taille de l´échantillon et éthique

Nous avons calculé et obtenu une taille minimale de l´échantillon de 117 donneurs de sang, au moyen de la formule de Lorentz:

$$N=\frac{Z^{2}PQ}{d^{2}}$$

avec p = 8,3% [10]. Dans le respect de l´éthique de la recherche, nous avons obtenu des autorisations de collecte de données des directeurs de HCY et CHDSMP ainsi qu´une clairance éthique N° 2019/01086/CEIRSH/MIM du Comité éthique institutionnel pour la recherche en santé humaine de l´Ecole des sciences de la santé de l´Université catholique d´Afrique centrale.

### Méthodologie

**Extraction de l´ADN:** cette extraction était faite à l´´Isocyanate de Guanidine. Dans un tube à Eppendorf, 200 μl d´Isocyanate de Guanidine était ajouté à 100 μl de plasma et l´ensemble était homogénéisé au vortex. Le mélange était incubé dans un mixeur thermostaté à 60°C pendant 10 min afin d´effectuer une lyse cellulaire complète. Après cette réaction, l´ADN d´intérêt était séparé du lysat par ajout de 250 μl d´isopropanol suivi d´une centrifugation à 15000 trs/min pendant 15 min. Le surnageant était jeté et le culot était mélangé à 1ml d´éthanol à 70% afin de permettre la précipitation de l´ADN, puis le mélange était centrifugé à 15000 trs/min pendant 5 min. L´éthanol était délicatement aspiré à l´aide d´une pipette et le tube était laissé à l´air libre pour évaporation complète de l´éthanol. L´ADN obtenu était solubilisé dans 50 μl d´eau distillée stérile et conservé à 0°C pour le test de PCR.

**Préparation du mélange réactionnel et amplification:** pour ce qui est de la première PCR, pour un volume réactionnel final de 25 μl par échantillon, 20 μl de master mix était préparé contenant: 12,9 μl d´eau distillée stérile; 2,5 μl de tampon EURx (10X); 2,5 μl de dNTP (4mM); 1 μl du mélange de chaque couple d´amorces externes (brin sens HBPr 134: 5´-TGC TGC TAT GCC TCA TCT TC-3´, brin antisens HBPr 135 : 5´-CAR AGA CAA AAG AAA AAT GG-3´) (25mM) ; 1μl de MgCl2 (25mM) et de 0,1 μl de Taq polymérase. Par la suite, 20 μl était introduit dans les micros tubes Eppendorf et 5 μl d´ADN obtenu par extraction était ajouté au mélange réactionnel portant alors le volume final à 25 μl dans chaque tube à PCR. Les tubes étaient enfin placés dans le thermocycleur (Applied Biosystems 2720 thermal Cycler) pour amplification selon le programme suivant:

Prédénaturation: 94°C, 3 minutes 30 (1 cycle); 2) Dénaturation: 94°C, 30 secondes (35 cycles); 3) Hybridation à 60°C, 30 secondes (35 cycles); 4) Extension à 72°C, 1,15 secondes (35 cycle); 5) Extension finale à 72°C, 4 minutes (1 cycle); 6) Conservation à 4°C, +∞.

A la différence de la première PCR, il était utilisé pour la deuxième PCR un couple amorces internes (HBPr 75: 5´-CAA GGT ATG TTG CCC GTT TGT CC-3´, brin antisens HBPr 94 : 5´-GGT AWA AAG GGA CTC AMG ATG-3´) et les produits de la première PCR comme ADN. Les amorces permettaient d´amplifier le gène Pol et de révéler les bandes d´ADN du VHB autour de 341 Pb.

**Electrophorèse sur gel d´agarose:** les amplicons issus de la deuxième PCR étaient analysés sur un gel d´agarose à 2%. Un marqueur de poids moléculaire (Trans 2K^®^ Plus DNA Marker) était inclus. La migration électrophorétique était faite dans un tampon TAE 1X à 120 volts pendant 30 mn et les amplicons ayant migré étaient visualisés grâce à un transilluminateur.

### Statistiques

La base de données était conçue sur tableur Excel 2019, et les différents tests statistiques étaient réalisés avec le logiciel d´accès libre R version 3.5.2 (2018-12-20) pour Windows version 10.0. Les tests statistiques utilisés pour la comparaison entre elles de plusieurs distributions observées étaient soit le test exact de Fisher, soit le test Chi2 de conformité au risque α = 0,05. La détermination des intervalles de confiance était réalisée à partir du calcul de la probabilité exacte basé sur la loi binomiale. Dans R, ce calcul est intégré dans le test Binomial exact que nous avons par conséquent utilisé pour lire les intervalles de confiance des pourcentages obtenus.

## Résultats

**Figure 1:**
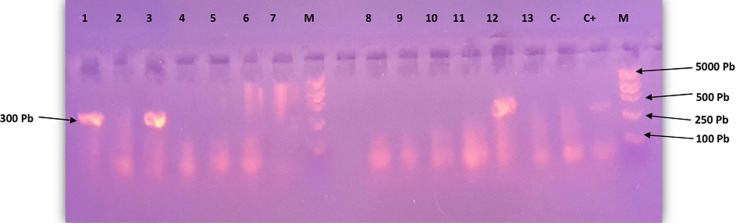
révélation des produits d´amplification sur gel d´agarose 2%

Durant la période de l´étude, 201 donneurs de sang étaient initialement enrôlés dont 118 à HCY et 83 au CHDSMP. Le sexe ratio H/F était de 6,7 (175 hommes et 26 femmes). L´âge moyen des participants était de 29,86 ans avec des extrêmes allant de de 18 à 55 ans. Le nombre moyen des dons de sang ayant déjà été effectués par les donneurs était de 1,79 avec des extrêmes allant de 1 à 10 dons. Des 201 donneurs, 08 étaient exclus de l´étude dont 03 (1,49%) étaient positifs au VIH, 01 (0,50%) à l´AgHBs, 01 (050%) au VHC et 03(1,49%) au tréponème et 193 donneurs potentiels ont été retenus. La recherche des marqueurs sérologiques du VHB (AcHBc, AgHBs, AcHBs, AgHBe et AcHBe) a permis d´obtenir 145/193 (75,13%) participants négatifs, 25 (12,95%) cas positifs à l´AcHBs, 03 (1,55%) cas positifs à AcHBc, AcHBs et AcHBe, 10 (5,18%) cas positifs à l´AcHBc et 09 (4,66%) cas positifs à l´AcHBc et AcHBs (Tableau 1). La recherche par PCR de l´ADN du VHB était effectuée sur 19/193 (9,83%) poches de sang contenant des marqueurs sérologiques AcHBc et/ou AcHBs, prédictifs de la présence du VHB; 3 cas étaient positifs (Figure 1) soit une prévalence à l´hépatite bbocculte de 15,79% [IC95% = 3,38-39,58]. Le risque résiduel était déterminé pour l´ensemble des 193 donneurs de sang éligibles au don, soit 1,55% (3/193) [IC95% =0,32-4,48] (Tableau 2).

**Tableau 1:** profil sérologique des marqueurs du virus de l´hépatite B

Marqueurs	AcHBc	AgHBs	AcHBs	AgHBe	AcHBe	Effectif
Profil	-	-	-	-	-	145
	+	-	-	-	-	10
	-	-	+	-	-	25
	+	-	+	-	-	09
	+	-	+	-	+	03
						193

**Tableau 2:** risque résiduel de l´hépatite B occulte

	Effectif	Prévalence (%)
Population d'étude	193	
Prévalence AcHBc +	10	5,18 [IC95% = 2,51 - 9,32]
Prévalence AcHBc + et AcHBs +	09	4,66 [IC95% = 2,15 - 8,67]
Prévalence AcHBc+/ADN VHB+	2	1,04 [IC95% = 0,13 - 3,69]
Prévalence AcHBc+ et AcHBs+/ADN VHB+	1	0,52 [IC95% = 0,13 - 2,85]
Risque résiduel de l´HBO		1,55 [IC95% = 0,32 - 4,48]

## Discussion

L´objectif de cette étude était de déterminer le risque résiduel de transmission du virus de l´hépatite B sous forme occulte par transfusion sanguine. Plus spécifiquement, il était question de déterminer la prévalence des anti-VHB (AcHBc et/ou AcHBs) chez les donneurs de sang éligibles au don dans deux structures sanitaires de la ville de Yaoundé, et le cas échéant, de détecter par PCR la présence de l´ADN du VHB dans les échantillons des donneurs de sang AcHBc et/ou AcHBs positifs. Les effectifs constitués au niveau des deux structures hospitalières étaient de 118 donneurs à l´HCY et 83 au CHDSMP avec un sex-ratio H/F de 6,7 (soit 6 hommes pour 1 femme) ce qui laisse penser que les femmes sont moins enclines aux dons de sang que les hommes. Cette faible participation des femmes au don pourrait s´expliquer par les conditions physiologiques telles que l´allaitement ou la grossesse.

Notre population regroupait deux types de donneurs, les donneurs volontaires (11) et les donneurs familiaux (190). Le type de don est une caractéristique importante à cause de son impact direct sur la sécurité transfusionnelle [11]. L´on en déduit que la population n´a pas la culture du don bénévole, d´où le nombre élevé de dons familiaux. Les patients demandeurs de sang sont des sujets à risque de contracter des infections transmises par voie parentérale. Dans notre étude, la prévalence des anticorps anti VHB était de 9,84% [IC95% = 6,03 % - 14,95%], représentant la proportion du risque de transmission du virus. Les prévalences obtenues en fonction du stade de l´hépatite étaient respectivement de 5,18% pour l´AcHBc isolé (caractéristique d´une hépatite B ancienne guérie), et de 4,66% pour l´AcHBc associée à l´AcHBs (caractéristique de l´immunité acquise). Cette prévalence est différente de celle rapportée par Biwole Sida *et al*. [9] au Cameroun, qui avait, au cours d´une étude similaire, obtenu une prévalence de l´AcHBc de 56,57%.

Nous avons vérifié par un test de χ^2^ (chi2) la conformité de nos résultats avec ceux de Biwole au risque α = 0,05, sous l´hypothèse nulle « Ho: il n´y a pas de différences significatives entre les prévalences ». La valeur seuil du χ^2^ pour α = 0,05 à 1 ddl est de 3,84. Cette valeur est largement dépassée, ce qui amène au rejet de Ho. On en déduit que sur la base de nos données, il existe un écart significatif par rapport à la prévalence de Biwole Sida (χ^2^ = 171,51, pour 1 ddl). Cette disparité des résultats s´expliquerait par une différence de prévalence du VHB dans la population d´étude des auteurs cités. En tenant compte du fait que notre étude a été réalisée des années après celle de Biwole Sida *et al*. [9], il est probable que l´écart observé traduirait une baisse de prévalence du portage de l´AcHBc et/ou AcHBs chez les donneurs de sang ces dernières années. Par contre, nos résultats sont comparables à ceux d’Ayed *et al*. [12] en Algérie qui étaient de 13,03% (χ^2^ = 1,73 < 3,84).

La détermination du risque résiduel de transmission du virus de l´hépatite B sous forme occulte nous a amené à rechercher son ADN au sein de nos donneurs catalogués comme étant potentiellement à risque d´infection. La prévalence obtenue était estimée à 1,55%, et se subdivisait en deux sous-groupes à savoir les sujets AcHBc+ isolés dont le taux de séropositivité était de 1,04 %, et les sujets AcHBc+ et AcHBs+ dont le taux de séropositivité était de 0,52%. Les valeurs ainsi obtenues ne sauraient expliquer la prévalence nationale car l´infection occulte par le VHB dépend de la population étudiée (donneurs de sang, hémodialysés, VIH+, VHC+ etc.). Elle est plus fréquente chez les patients atteints d'une maladie hépatique chronique (exclus de notre étude), et moins fréquente parmi les donneurs de sang en bonne santé [13]. Néanmoins, elle est comparable à la prévalence de 2,4% (χ^2^ = 0,59 < 3,84) trouvée par Morales-Romero *et al*. [14] au sein d´un échantillon de donneurs de sang (population à bas risque) en Amérique latine. Aussi, au regard de ce qui précède, nous pouvons affirmer que notre échantillon présente des risques certains de réactivation virale en cas de transfusion à un patient soumis à un traitement immunosuppresseur. En effet, l´HBO étant associée à une suppression de l´activité virale par le système immunitaire, les personnes immunodéprimées sont particulièrement à risque de développer une réactivation du VHB [14].

Nous avons constaté que les échantillons testés positifs à l´ADN du VHB dans notre population provenaient de donneurs guéris d´une hépatite ancienne. Cette observation rejoint celle de Kwak & Kim [6] qui ont montré que la réponse immunitaire au virus de l´hépatite B reste positive longtemps après une infection aiguë et en plus, l´ADN dudit virus peut être détecté par PCR dans le sérum, plus d'une décennie après une guérison apparente à l´infection. Nos résultats montrent que le taux de détection de l´ADN du VHB était plus élevée chez les sujets AcHBc+/AcHBs- (1,04 [IC95% = 0,13 - 3,69]), intermédiaire chez les sujets AcHBc+ et AcHBs+ (0,52 [IC95% = 0,13 - 2,85]) et plus faible chez les sujets séronégatifs (aucun cas positif détecté), ce qui s´apparente aux observations de Bréchot *et al*. [15]. Les sujets AcHBc+/AcHBs- (1,04%) ont probablement un faible taux d´infection au VHB dû à l´indétectabilité de l´AgHBs, les personnes AcHBc+ et AcHBs+ (0,52%) auraient été guéries d'une infection antérieure, mais présentent une persistance du VHB à des niveaux bas, tandis que les personnes séronégatives semblent n´avoir jamais été en contact avec le virus du VHB car ne possédant aucun marqueur sérologique de l'infection.

Les éléments évoqués ci-dessus apportent la preuve de l´existence du VHB occulte dans notre population de départ. Ladite infection a été détectée sur des sujets montrant des signes d´une hépatite B ancienne guérie (AcHBc+) et des sujets ayant acquis l´immunité (AcHBc + et AcHBs +). On en déduit donc que conformément à Conjeevaram *et al*. [13], l´infection occulte est associée au maintien, par le système immunitaire, du VHB à des niveaux très bas dans l´organisme. En définitive, notre étude confirme le risque résiduel de transmission par transfusion de l´infection B occulte en l´absence de dépistage des anticorps anti-VHB (AcHBc et/ou AcHBs). Ce risque a été de 1,55% [IC95% = 0,32 - 4,48].

## Conclusion

Il ressort de cette étude qu´un risque de transmission par transfusion de l´infection de l´HVB subsiste chez les donneurs de sang malgré l´éligibilité des poches de sang à l´issu des tests de qualification biologique des dons. Ce risque appelle l´attention du corps médical sur l´importance de la recherche des marqueurs sérologiques AcHBc et/ou AcHBs du VHB lors de la qualification biologique des dons.

### Etat des connaissances sur le sujet

- La transmission par transfusion du VHB est réelle dans les pays ou la recherche de l´AgHBs et du génome viral n´est pas appliquée sur les dons de sang et d´organes.

### Contribution de notre étude à la connaissance

- La prévalence de l´hépatite B occulte est de 15,79% dans les banques de sang de HCY et CHDSMP;
- Le risque résiduel de transmission par transfusion de l´infection à VHB y est de 1,55%.

## Remerciements

Les auteurs adressent leurs remerciements aux participants, aux personnels des banques de sang de HCY et CHDSMP et aux directions des hôpitaux.
